# Synergistic Strategies for Castration-Resistant Prostate Cancer: Targeting AR-V7, Exploring Natural Compounds, and Optimizing FDA-Approved Therapies

**DOI:** 10.3390/cancers16162777

**Published:** 2024-08-06

**Authors:** Muntajin Rahman, Khadija Akter, Kazi Rejvee Ahmed, Md. Maharub Hossain Fahim, Nahida Aktary, Moon Nyeo Park, Sang-Won Shin, Bonglee Kim

**Affiliations:** 1 Department of Pathology, College of Korean Medicine, Kyung Hee University, Seoul 02447, Republic of Korea; muntajinrahman899@gmail.com (M.R.); kazirejveeahmed@gmail.com (K.R.A.); mehrub.btgeiu@gmail.com (M.M.H.F.); nahidabph@gmail.com (N.A.); mnpark@khu.ac.kr (M.N.P.); 2 Department of Plasma Bio Display, Kwangwoon University, Seoul 01897, Republic of Korea; santaafrin02@gmail.com; 3 Department of Humanities & Social Medicine, School of Korean Medicine, Pusan National University, 49 Busandaehak-ro, Mulgeum-eup, Yangsan-si 50612, Republic of Korea

**Keywords:** castration-resistant prostate cancer, androgen receptor splice variant 7, androgen receptor blockers, natural compounds, herbal medicine

## Abstract

**Simple Summary:**

Castration-resistant prostate cancer (CRPC) resists standard therapies, partly due to the androgen receptor variant AR-V7. Conventional treatments like chemotherapy and radiation have limited success and significant side effects. Natural compounds from herbal medicine offer promise as complementary therapies. This review examines how these natural compounds can improve treatment outcomes and reduce side effects, presenting a safer and more effective approach to managing CRPC and overcoming resistance to conventional therapies.

**Abstract:**

Castration-resistant prostate cancer (CRPC) remains a significant therapeutic challenge due to its resistance to standard androgen deprivation therapy (ADT). The emergence of androgen receptor splice variant 7 (AR-V7) has been implicated in CRPC progression, contributing to treatment resistance. Current treatments, including first-generation chemotherapy, androgen receptor blockers, radiation therapy, immune therapy, and PARP inhibitors, often come with substantial side effects and limited efficacy. Natural compounds, particularly those derived from herbal medicine, have garnered increasing interest as adjunctive therapeutic agents against CRPC. This review explores the role of AR-V7 in CRPC and highlights the promising benefits of natural compounds as complementary treatments to conventional drugs in reducing CRPC and overcoming therapeutic resistance. We delve into the mechanisms of action underlying the anti-CRPC effects of natural compounds, showcasing their potential to enhance therapeutic outcomes while mitigating the side effects associated with conventional therapies. The exploration of natural compounds offers promising avenues for developing novel treatment strategies that enhance therapeutic outcomes and reduce the adverse effects of conventional CRPC therapies. These compounds provide a safer, more effective approach to managing CRPC, representing a significant advancement in improving patient care.

## 1. Molecular Mechanisms Driving Castration-Resistant Prostate Cancer Cell

An advanced kind of prostate cancer known as castration-resistant prostate cancer (CRPC) develops when regular androgen deprivation treatment (ADT) is ineffective in treating the malignancy. A wide range of intricate genetic and molecular alterations that are controlled by intricate signaling networks and cellular adaptations are the cause of this resistance within the tumor cells [1]. Prostate cancer cells depend on androgens for growth and survival, with the androgen receptor (AR) being pivotal in mediating androgen-dependent transcriptional processes [2]. In CRPC, cancer cells develop mechanisms to bypass the need for ligand binding to the androgen receptor (AR), rendering them unresponsive to traditional hormone therapies [3]. Several mechanisms contribute to this resistance, including amplification and overexpression of the AR gene, point mutations leading to ligand-independent AR activity, and alterations in co-regulatory proteins that modify AR function [4]. Additionally, CRPC cells often activate alternative signaling pathways to sustain their growth and survival. For instance, prostate cancer frequently exhibits disruption of the PI3K/Akt/mTOR pathway, which is independent of androgen receptor (AR) signaling and promotes cell survival and proliferation. Furthermore, an increase in tumor aggressiveness and treatment resistance is associated with aberrant activation of the Wnt/β-catenin pathway, which is connected to the evolution of CRPC. Androgen receptor splice variants (AR-Vs), such as AR-V7, are developing, and this is a major resistance mechanism in CRPC [5]. These variants lack the ligand-binding domain of the full-length AR, remaining constitutively active and driving tumor growth in the absence of androgens [6]. AR-V7, in particular, is linked to resistance against both traditional ADT and newer anti-androgen therapies, presenting substantial challenges in CRPC treatment [5]. Moreover, intra-tumoral androgen biosynthesis has been identified as a critical factor in CRPC progression. Prostate cancer cells upregulate enzymes that convert precursor molecules into androgens within the tumor microenvironment, thereby maintaining a local supply of androgens that fuels tumor growth even when systemic androgen levels are low [6]. CRPC is characterized by complex genetic, molecular, and cellular changes that enable tumor cells to withstand androgen deprivation. Understanding the nuances of these mechanisms is essential for developing novel therapeutic strategies aimed at targeting the diverse factors contributing to CRPC resistance, ultimately improving outcomes for patients with advanced prostate cancer [7].

## 2. Global Statistics on the Incidence and Mortality of Prostate Cancer

Prostate cancer is a leading malignancy among men globally. In 2020, approximately 1.4 million new cases were reported, making it the second most common cancer in men after lung cancer [8]. Projections estimate that by 2040, the incidence will rise to over 2.3 million new cases with 740,000 deaths, primarily due to population growth and aging [9]. Key risk factors include older age, African ancestry, and family history. Additional factors linked to increased risk are high body fat, tall stature, high dairy and calcium intake, and low plasma selenium levels. Elevated alpha-tocopherol levels have also been associated with a higher risk of prostate cancer [10]. Since the introduction of prostate-specific antigen (PSA) testing in 1986, the incidence of prostate cancer in the US and Europe spiked and subsequently declined due to issues like false positives, overdiagnosis, and overtreatment, affecting up to 20–50% of diagnosed men who might never have developed symptoms [11]. Prostate cancer incidence is highest in regions with high Human Development Index (HDI) scores, such as Northern and Western Europe and Australia/New Zealand, with rates around 86.4 per 100,000 men. Conversely, South Central and South-Eastern Asia report the lowest incidence rates at about 5.0 per 100,000. The disparity in incidence rates is partly due to varying accessibility to PSA testing [12].

Racial differences in prostate cancer incidence and progression are increasingly evident [13,14]. Black men face significantly higher incidence and mortality rates compared to other racial groups [15,16]. These disparities are attributed to a complex interplay of socioeconomic factors, genetic and molecular differences, and variances in risk factors [17]. Population-level studies have identified these discrepancies [14,18], yet clinical trials often underrepresent Black men, accounting for less than 3% of global trial participants, thus not reflecting real-world demographics accurately [19,20]. Addressing these disparities requires a multifaceted approach, including increased representation in clinical trials, better understanding of genetic and biological differences, and addressing socioeconomic factors. Enhanced research and targeted interventions can help mitigate the higher risks faced by specific populations, ultimately improving outcomes for all men diagnosed with prostate cancer [21].

## 3. The Role of Androgen Receptor Variants and Coactivators in Prostate Cancer Progression

The Androgen Receptor (AR) is crucial for the normal functioning of prostate tissue, overseeing development, differentiation, and homeostasis by balancing cell proliferation and apoptosis [18]. It mediates androgen effects, regulates the expression of prostate-specific antigen (PSA) and seminal fluid components, and modulates cellular signaling with growth factors and cytokines [18,22]. AR facilitates communication between epithelial and stromal cells, maintains structural integrity [23], and ensures immune-privileged status in the prostate against inflammation and autoimmunity, essential for prostate gland physiology [24].

In prostate cancer, AR splice variants are produced through alternative splicing, where differential processing of AR pre-mRNA removes exons encoding the ligand-binding domain (LBD) [25,26]. Mutations or changes in splicing factors and the spliceosome machinery influence this splicing [27]. The resultant AR protein isoforms lack the LBD but retain the N-terminal domain (NTD) and DNA-binding domain (DBD) [28]. These constitutively active variants can bind to androgen response elements (AREs) in DNA and activate target genes without androgens, enhancing cancer cell survival and proliferation. This leads to castration-resistant prostate cancer (CRPC), where the cancer progresses despite androgen deprivation therapy (ADT), contributing to therapy resistance and disease advancement [29].

**Figure 1:**
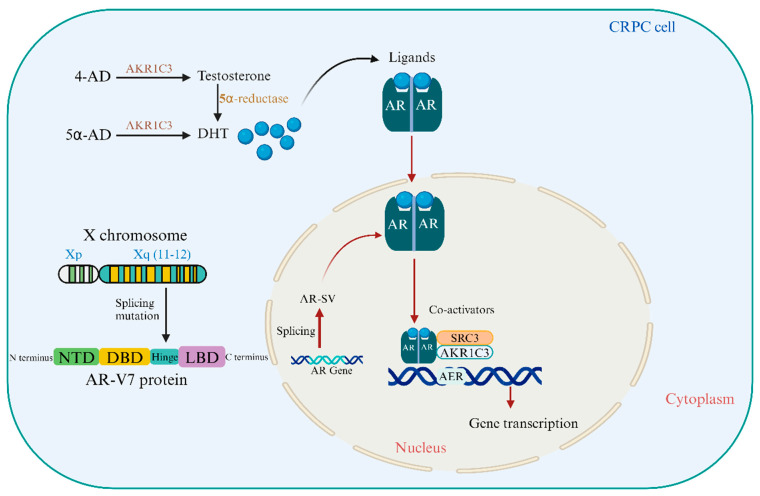
Structural and Functional Insights into the Androgen Receptor and Its Role in CRPC. The androgen receptor (AR) gene, located on the X chromosome at Xq11-12, encodes the AR-V7 protein, which includes four distinct domains: N-terminal domain (NTD), DNA-binding domain (DBD), hinge region, and ligand binding domains (LBD). Activation of the AR by androgens such as testosterone and dihydrotestosterone (DHT) initiates a cascade of events that promote prostate cancer cell proliferation. Aldo-keto reductase family 1 member C3 (AKR1C3) is overexpressed in castration-resistant prostate cancer (CRPC) and is crucial for the synthesis of DHT from weak androgens, contributing to the persistence of AR signaling in low-androgen environments. Furthermore, testosterone is converted to the more potent androgen DHT through the action of 5α reductase, further fueling the growth of CRPC cells despite androgen deprivation therapy.

Antioxidants regulate AR and AR-V7 expression and activity, influencing CRPC development and therapy resistance. They also protect cancer cells from oxidative damage, which can affect treatment outcomes. Understanding these pathways is crucial for developing CRPC-specific therapeutics and improving patient outcomes. Figure 1 illustrates the structural domains of the AR protein and the biochemical pathways involving AKR1C3 and 5α-reductase that sustain androgen receptor activation and CRPC progression.

### 3.1. The Crucial Strategies That Can Target AR-V7 for the Treatment of CRPC

CRPC is resistant to androgen deprivation therapy due to AR-V7, requiring novel techniques to target AR-V7 directly or indirectly to improve treatment results [30]. Modern tactics and ongoing investigations in this quickly evolving field are discussed in this discussion of AR-V7 strategies.

1. AR-V7 Resistance: Two antisense oligonucleotides (AONs) were created to target AR pre-mRNA splicing enhancers, restoring sensitivity to androgen deprivation and inducing apoptosis in CRPC cell lines [31]. AKR1C3 and AR-V7 interact in prostate rebiopsy tissues, preventing degradation and repressing B4GALT1, a prostate cancer tumor suppressor gene essential for CRPC cell proliferation post-androgen deprivation [32].
2. Targeting AR-V7 Translation/Transcription: AR-FL binds to ARBS2, an AR intron 2 enhancer, to inhibit chromatin. These negative autoregulatory mechanisms of ADT enhance AR-FL and AR-V mRNA simultaneously. The AR-FL protein boosts AR-FL and AR-V transcripts after ADT [33]. A tiny molecule SC912 interacts with full-length AR and AR-V7 via its N-terminal domain. Pan-AR targeting uses AR-NTD amino acids 507–531. SC912 prevented AR-V7 nuclear localization, DNA binding, and transcription. SC912 stopped AR-V7-positive CRPC cell growth, cell-cycle arrest, and death. SC912 inhibited AR signaling and CRPC xenograft development in AR-V7-expressing cells. These findings suggested SC912 could treat CRPC [34].
3. Combination Therapies: New targeted, immunological, AR-targeting, and chemotherapy. Metastatic Castration-Resistant Prostate Cancer Treatment) and AFFINITY (Cabazitaxel/Prednisone Alone or With Custirsen for 2nd-line Prostate Cancer Chemotherapy). Reactivating AR activity is another docetaxel resistance mechanism. A gene mutation that makes AR translocation independent of microtubule control can produce docetaxel resistance. Drug interactions with cytotoxic treatment can cause fatal neutropenic enterocolitis [35].
4. AR-V7-Targeted Immunotherapy: AR-V7-Targeted Immunotherapy (MVI-118) is a DNA vaccine for metastatic prostate cancer, promoting a CD8+T cell-mediated immune response against AR-overexpressing cancer cells. Genomic profiling can detect genetic changes and molecular subtypes, potentially predicting treatment response. Next-generation sequencing technologies can also reveal immune response landscapes and immunotherapy-predictive biomarkers in tumor microenvironments [5,36].
5. Epigenetic Modulators: Emerging evidence links non-coding RNAs to CRPC development and treatment resistance, while AR-epigenetic pathways are specifically engaged in PC carcinogenesis [7]. Histone regulators including LSD1 and EZH2 can have a significant impact on AR expression and help CRPC re-programme AR activation. The expression of EZH2 and BRD4, which stimulates regulators and engages with acetylated histones to coactivate AR. Epigenetic alterations help PCa cells respond to AR signaling suppression. Recent research conducted that EZH2 can make CRPC tumors resistant to DNA-damaging therapies
6. Proteolysis-Targeting Chimeras (PROTACs): In recent years, targeting AR protein for breakdown by AR-targeted proteolysis targeting chimeras (PROTACs) or small-molecule degraders has become an interesting and potentially useful way to treat metastatic CRPC (mCRPC) [37]. The way AR expression inhibitor functions are different from how PROTAC targets AR protein. AR expression inhibitor uses antisense oligonucleotides to target AR mRNA by binding the complementary region of AR mRNA, which significantly lowers mRNA production [38].

### 3.2. Certain Processes Supporting CRPC Evolution Similar to AR-V7

Various mechanisms confer resistance to next-generation AR-directed therapies, such as activation of canonical AR-signaling through AR amplification, feedback pathway activation (e.g., AKT/mTOR/PI3K), and AR mutations or substitutions (e.g., AR splice variants) [39].

Epigenetic modifications also play a significant role in cancer progression. These changes can provide insights into early cancer detection, prognosis, and risk assessment. Epigenetics involves heritable variations in gene expression without altering the DNA sequence [40]. mRNA acts as a link between genetic information and protein synthesis, making it crucial in converting genotypes into phenotypes [41]. Epigenetic changes can occur in both enhancer regulatory and promoter regions around the transcription start point, impacting transcription, DNA repair, and replication through modifications like phosphorylation, acetylation, methylation, and ubiquitination. DNA methylation has been the most studied regulatory method, but histone protein modifications are now recognized as significant chemical components of epigenetic up-regulation. Research in Clonal Hematopoiesis of Indeterminate Potential (CHIP) has shown that most mutations occur in pre-leukemic driver genes, affecting other illnesses beyond hematologic malignancies [42].

Epigenetic modifications, similar to AR-V7, contribute to CRPC development. DNA methylation and histone modifications are the most common epigenetic changes in prostate cancer, affecting gene expression and promoting cancer progression [43]. Key epigenetic modifications include RNA methylation, DNA methylation, histone modifications, non-coding RNAs, and chromatin remodeling, which regulate solid tumors and hematologic malignancies [44].

Given these insights, AR-V7 is gaining attention as a resistance mechanism to anti-androgen receptor therapies for CRPC and as a potential predictive marker for these therapies. Developing next-generation drugs targeting AR-V7 signaling is critically necessary [45].

### 3.3. Clinical Intervention Linked to AR-V7 in the Development of CRPC

#### 3.3.1. AR Neutral CRPC

Androgen-targeted therapies have long been the cornerstone of prostate cancer treatment. However, targeting AR often leads to drug resistance. An alternative approach involves the loss of AR function. Various prostate epithelial cell types, including basal, stem, and neuroendocrine cells, do not express AR [46]. The expansion of these AR-negative cell populations, flexible differentiation states, and AR loss due to therapeutic pressures can result in tumors inherently resistant to AR antagonism. Although several studies have noted AR low/negative expression patterns, their predominance has often been overlooked [47].

#### 3.3.2. Neuroendocrine Prostate Cancer (NEPC)

NEPC, a predominant subgroup of AR-negative CRPC, is characterized by the upregulation of neuroendocrine differentiation markers such as synaptophysin and chromogranin A, alongside the downregulation of AR expression. In normal prostate tissue, neuroendocrine cells are present in small numbers; approximately 1% of primary prostate cancer cases are NEPC. However, in CRPC, this differentiation can increase to 30%, which is associated with poor clinical outcomes [47].

#### 3.3.3. AR Regulation in AR-Independent CRPCs

Despite the well-documented mechanisms of AR-driven CRPC recurrence, AR-negative CRPC subtypes are becoming more prevalent, necessitating an understanding of AR regulation in this context. Mechanisms leading to AR downregulation include ubiquitin-mediated degradation, post-transcriptional targeting by microRNAs and RNA-binding proteins, and promoter methylation. Understanding these mechanisms is crucial for addressing the emergence of AR-low CRPC subtypes [47].

## 4. Androgen Receptor Splice Variants AR-V7 in CRPC Prostate Cancer

Over 20 different androgen receptor variants (AR-Vs) have been identified in prostate cancer cell lines and biopsy specimens of CRPC [48]. AR-Vs in CRPC primarily result from AR gene rearrangements and alternative splicing of AR mRNA. These AR-Vs can be categorized into three groups based on their transcriptional activity: dormant AR-Vs (e.g., AR-V13, AR-V14), selectively active AR-Vs (e.g., AR-V1, AR-V9), and constitutively active AR-Vs (e.g., AR-V7, ARv567es). AR-V7, in particular, has been the focal point of extensive clinical research due to its significant role in CRPC progression [48].

AR-Vs, including AR-V7, lack the AR ligand-binding domain (LBD) but retain the AR transcriptional activation domain and DNA-binding domain (DBD), allowing them to regulate transcription independently of androgen binding. Consequently, AR-Vs are resistant to conventional and next-generation hormonal therapies that target the AR-LBD. Structurally, AR-V7 includes exons 1, 2, and 3 encoding the N-terminal domain (NTD), followed by a cryptic exon [49]. In CRPC, AR-V7 expression is unique and inversely regulated by signals from the full-length androgen receptor (AR-FL). AR-V7 can facilitate the development of a mature phenotype when the canonical signaling pathway is blocked [50,51,52].

Both AR-V7 and AR-FL bind to chromatin, but AR-V7 interacts with distinct binding sites and various repressors and coregulators. ARv567es, another significant variant, lacks exons 5, 6, and 7, retaining only the C-terminal portion encoded by exon 8. In CRPC, AR-V7 and ARv567es have been detected in whole blood samples; however, AR-V7 is typically absent in hormone-naïve prostate cancer [53]. The AR-V7 protein can enter the nucleus of prostate cancer cells and remain constitutively active without ligand interaction. While AR-V7 is frequently observed in CRPC specimens, it is rarely detected in hormone-naïve prostate cancer [54].

**Figure 2:**
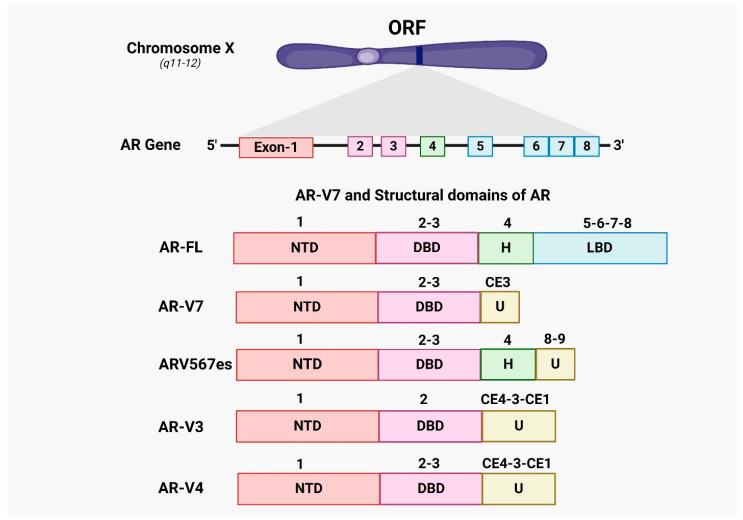
Overview of AR-V7 and AR splice variants. The androgen receptor (AR) gene, located on the X chromosome, comprises eight exons that encode four primary domains: The N-terminal domain (NTD), DNA-binding domain (DBD), hinge region (H), and ligand-binding domain (LBD). The AR gene can undergo alternative splicing, producing several splice variants, each with distinct structures and functions. In the full-length androgen receptor (AR-FL), exons 1–8 encode the complete set of domains. However, alternative splicing can involve cryptic exons (CE1-4) and exon 9, generating a unique sequence (U) not found in AR-FL. AR-V7, also known as AR3, is a significant splice variant that terminates at the end of exon 3, lacking the LBD, and includes 16 unique amino acids from cryptic exon 3 (CE3). This modification results in AR-V7’s constitutive activity, allowing it to activate AR signaling pathways without the need for androgen binding. Additionally, other constitutively active AR splice variants, such as ARv567es, AR-V3, and AR-V4, are described. Each variant arises from different exon combinations, producing unique open reading frames (ORFs) that encode their respective receptor proteins.

Nevertheless, in cases of hormone-naive prostate cancer, the increased levels of AR-V7 were strongly associated with biochemical recurrence (BCR) after prostatectomy. Reducing the expression of AR-V7 using shRNA in xenografts and cell lines of CRPC resulted in decreased cell growth under conditions of androgen deprivation [54]. AR-V7, which does not possess the ligand-binding domain, is anticipated to serve as a main defense against androgen receptor signaling inhibitors (ARSI), such as CYP17 inhibitors (abiraterone) and next-generation androgen receptor antagonists (enzalutamide, apalutamide, and darolutamide). Figure 2 highlights the structural differences between AR-FL and its splice variants, emphasizing the functional implications of these alternative splicing events in castration-resistant prostate cancer (CRPC).

## 5. Adaptive Pathways and Treatment Options in CRPC Prostate Cancer

Over the past two decades, various treatments have demonstrated efficacy in managing metastatic castration-resistant prostate cancer (mCRPC). The range of treatment strategies for mCRPC is expanding, with both broad-spectrum and tailored therapies anticipated to improve overall survival rates in the coming decade. Docetaxel, introduced in 2004, was the first treatment to significantly enhance survival rates for mCRPC patients [55]. However, many patients eventually develop resistance to docetaxel, limiting its long-term effectiveness. To address this limitation, the US Food and Drug Administration (FDA) has approved five new agents for mCRPC treatment since 2010: abiraterone acetate, enzalutamide, cabazitaxel, radium-223, and sipuleucel-T. Despite their benefits, these agents are not without limitations. Patients can develop resistance to these treatments over time, and they often come with significant side effects [56]. For example, abiraterone acetate and enzalutamide are often administered with steroids like prednisone to mitigate adverse effects such as pain, inflammation, nausea, and allergic reactions, and to manage testosterone levels to prevent tumor growth [57]. This section highlights the FDA-approved drugs targeting mCRPC, focusing on their limitations and the ongoing challenges in overcoming resistance and managing side effects, as detailed in Table 1.

### 5.1. First-Generation Chemotherapy for Prostate Cancer

The development and FDA approval of antiandrogen therapies have significantly transformed the treatment landscape for prostate cancer, particularly in managing castration-resistant prostate cancer (CRPC). Over the past decade, advancements in understanding the molecular mechanisms of CRPC have led to significant improvements in its treatment [68]. First-generation therapies for prostate cancer comprise bicalutamide, mitoxantrone, cabazitaxel, and docetaxel. This section highlights the classification of chemotherapeutic drug and mechanism of action in the treatment of Prostate cancer, as detailed in Table 2.

#### 5.1.1. Docetaxel

**Figure 3:**
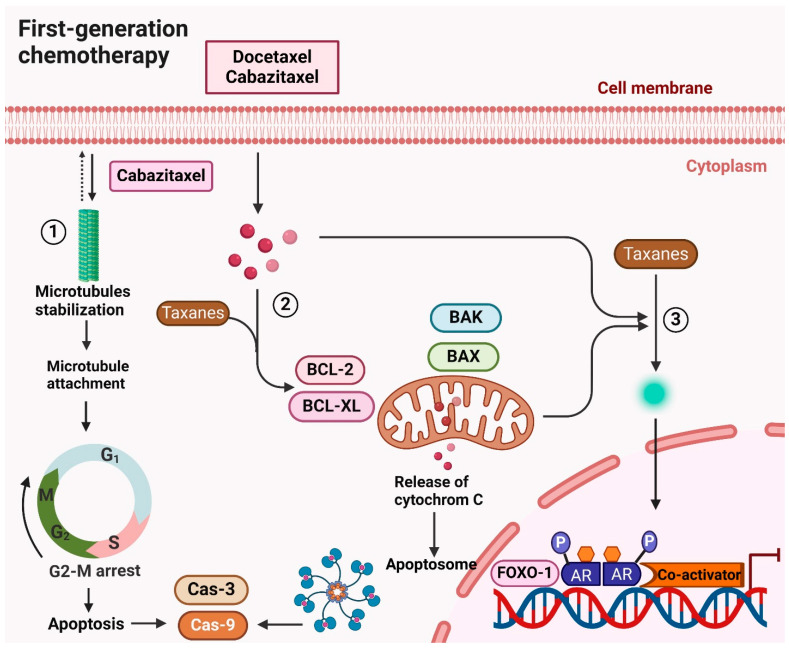
Mechanism of action of first-generation chemotherapy (Docetaxel, cabazitaxel) in treating CRPC. Docetaxel and cabazitaxel, both taxane-based chemotherapeutic drugs, exert their anticancer effects primarily by targeting microtubules. These drugs bind to β-tubulin, stabilizing microtubules and preventing their proper assembly. This disruption inhibits the G2-M phase transition of the cell cycle, leading to cell cycle arrest and apoptosis. Additionally, taxanes inhibit androgen receptor (AR) transcriptional activity by blocking FOXO1-mediated AR function, which is crucial in treating castration-resistant prostate cancer (CRPC). By obstructing AR signaling, taxanes effectively disrupt the growth and survival mechanisms of CRPC cells. Taxanes also promote apoptosis through the activation of pro-apoptotic proteins such as BAK and BAX while inhibiting anti-apoptotic proteins like BCL-2 and BCL-XL. This dual action triggers the intrinsic apoptotic pathway, characterized by the release of cytochrome c from mitochondria, culminating in cell death. Overall, the combination of microtubule stabilization, inhibition of AR signaling, and promotion of apoptosis underscores the therapeutic efficacy of docetaxel and cabazitaxel in managing CRPC.

In recognition of its anticancer attributes, the taxane-based chemotherapy medication docetaxel is used to treat prostate cancer. It causes cell death by preventing the formation of microtubules during both interphase and mitosis (Figure 3) [85]. Even though the FDA approved it in 2004 for CRPC when combined with prednisone, the improvement in overall survival was only noticeable for two to three months [55,86]. Patients with post-docetaxel CRPC had no further alternatives for therapy before 2010 [22]. For 10 rounds, docetaxel is normally infused intravenously once every three weeks, with dose adjustments made by patient tolerance. It can, however, have serious side effects, including cytopenia, nausea, vomiting, and neutropenic sepsis, just like other chemotherapy medications [87].

#### 5.1.2. Cabazitaxel

Cabazitaxel, a taxane derivative, was approved by the FDA in 2010 as the first post-docetaxel therapy for metastatic CRPC, leading to a 2.4-month survival improvement [88]. It shows anticancer efficacy in patients with tumors resistant to both docetaxel and post-docetaxel treatment, overcoming taxane resistance [89]. As a therapy alternative, cabazitaxel and prednisone are combined. By stabilizing microtubules, limiting AR’s nuclear translocation and lowering its transcriptional activity, taxane chemotherapy drugs like cabazitaxel block AR signaling [90]. In clinical trials, cabazitaxel demonstrated antitumor efficacy in cases where docetaxel, abiraterone, or enzalutamide was ineffective. This success is attributed to its ability to suppress AR variants (AR-Vs) that can bypass taxane resistance [91]. Figure 3 illustrates the molecular mechanisms by which docetaxel and cabazitaxel combat CRPC. Despite their benefits, the limitations of these drugs, including resistance development and significant side effects, highlight the ongoing challenges in effectively treating CRPC.

#### 5.1.3. Mitoxantrone

Mitoxantrone is a synthetic drug used as a second-line chemotherapeutic agent for treating prostate cancer (PC). It induces immunogenic cell death in PC cells by activating eukaryotic initiation factor 2 [92]. However, a retrospective analysis of several phase 3 mitoxantrone studies indicated that while some patients experienced symptomatic improvement, there were no significant survival benefits. Common side effects included fatigue, dyspnea, and pancytopenia [93].

### 5.2. Androgen Receptor Blockers

**Figure 4:**
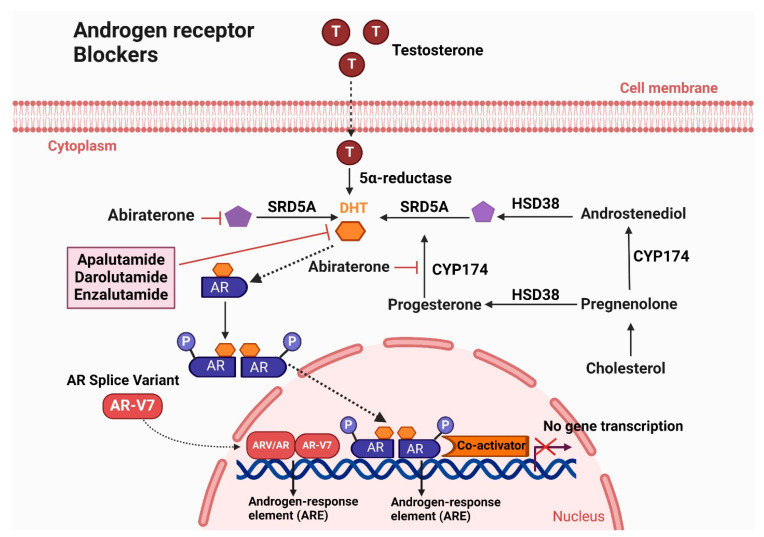
Mechanism of action of androgen receptor blockers (Apalutamide, Darolutamide, and Enzalutamide) in treating CRPC. Enzalutamide, Apalutamide, and Darolutamide are androgen receptor (AR) antagonists that effectively inhibit the androgen signaling pathway, crucial for the progression of castration-resistant prostate cancer (CRPC). Enzalutamide binds to the AR, preventing testosterone from attaching to the receptor. This inhibition blocks the translocation of the AR into the cell nucleus, thereby preventing the activation of AR target genes that promote cancer cell growth. In addition to direct AR antagonism, Abiraterone acetate targets androgen synthesis. It inhibits the enzyme 17α-hydroxylase and C17,20-lyase, which are essential for androgen production, by blocking their activity on the CYP-17. This action significantly reduces the levels of testosterone and other androgens, preventing their binding to the AR. Apalutamide and Darolutamide function similarly to Enzalutamide by binding to the AR and preventing its activation by testosterone. This inhibition blocks AR-mediated transcriptional activity, thereby hindering the growth of CRPC cells. By disrupting the androgen signaling pathway through these different mechanisms, these treatments aim to reduce AR activity and slow the progression of CRPC.

Androgen suppression therapy, also known as novel hormone therapy, targets the androgen signaling pathway to reduce androgen levels [94]. Overexpression of androgens drives the progression of both metastatic hormone-sensitive prostate cancer (mHSPC) and metastatic castration-resistant prostate cancer (mCRPC) [95]. Antiandrogens primarily inhibit androgen receptor (AR) signaling by preventing its nuclear translocation and subsequent activation of AR-targeted genes. The FDA has approved several drugs, including enzalutamide, apalutamide, and darolutamide. Figure 4 illustrates the Mechanism of action of androgen receptor blockers (Apalutamide, Darolutamide, and Enzalutamide) in treating CRPC.

#### 5.2.1. Enzalutamide

Enzalutamide, also known as MDV3100, is the first FDA-approved second-generation AR antagonist designed for treating CRPC. It has a significantly higher affinity for AR binding compared to first-generation AR antagonists [96]. Enzalutamide competitively binds to the ligand-binding domain (LBD) of AR, blocking androgen binding, nuclear translocation, DNA binding, and co-activator recruitment [97,98]. The FDA approved enzalutamide for mCRPC in 2012, for non-metastatic CRPC (nmCRPC) in 2018, and for castration-sensitive prostate cancer (CSPC) in 2019 due to its significant prolongation of castration-resistant free survival time [99]. However, its high steady-state brain levels have been associated with CNS-related events, including seizures, due to antagonism of the GABAα receptor [96,100,101].

#### 5.2.2. Apalutamide

Apalutamide (ARN-509), developed with a lower steady-state brain level, reduces the incidence of seizure side effects compared to enzalutamide [102]. It shares a similar core structure with enzalutamide and also acts as a full AR antagonist with high binding affinity to the AR LBD [102]. Apalutamide significantly extends metastasis-free survival in non-metastatic CRPC and overall survival in metastatic CSPC [103,104]. Both enzalutamide and apalutamide inhibit various stages of AR-mediated transcription, including competition with DHT for AR binding, prevention of AR nuclear translocation, and obstruction of DNA binding and cofactor recruitment [102].

#### 5.2.3. Darolutamide

Darolutamide, a novel AR antagonist, was developed to prevent AR nuclear translocation. In competitive AR binding experiments, darolutamide and its active metabolite (ORM-15341) showed significantly higher potency than enzalutamide and apalutamide, with lower Ki and IC50 values [105]. Darolutamide and ORM-15341 effectively inhibit both wild-type AR and clinically significant AR mutations [105]. This comprehensive suppression of AR mutations is a significant advancement in prostate cancer treatment, potentially reducing treatment resistance [106]. Additionally, darolutamide demonstrated minimal blood-brain barrier penetration and reduced in vivo growth of CRPC xenografts in rats and mice [105,107]. Figure 4 illustrates the mechanisms by which androgen receptor blockers—apalutamide, darolutamide, and enzalutamide—function to treat castration-resistant prostate cancer (CRPC). These drugs target and inhibit AR signaling pathways, reducing the progression and impact of CRPC.

### 5.3. Androgen Biosynthesis Inhibitors

Instead of primarily blocking the androgen receptor, another method of inhibiting androgen signaling is to target the upstream synthesis of androgens. The cytochrome P450 17α-hydroxylase-17,20-lyase (CYP17A1) enzyme is essential for the generation of androgens in the testes, adrenal glands, and malignancies. The testosterone production pathway is interfered with by blocking CYP17A1, which lowers androgen levels and stops the formation of dihydrotestosterone (DHT).

#### Abiraterone Acetate

The first of the second-generation antiandrogens, abiraterone acetate, was approved by the FDA in April 2011 (Table 1). Unlike standard antiandrogens that target the androgen receptor, it is still the only FDA-approved inhibitor of androgen production for the treatment of prostate cancer (PC). Abiraterone acetate, a very potent and specific inhibitor of CYP17A1, prevents men with metastatic castration-resistant prostate cancer (mCRPC) from producing androgens [108,109]. This dual inhibition of androgen production in the testes and adrenal glands makes abiraterone acetate more effective than Gonadotropin-releasing hormone (GnRH) analogs (ADT) in androgen-dependent prostate cancer [110]. Despite its defined survival advantage in treating mCRPC, abiraterone acetate causes significant toxicity due to non-specific inhibition of other CYP family members. To address this issue, researchers developed new compounds with structural variations to reduce side effects caused by interactions with steroidal hormones. These new compounds include non-steroidal cores, offering the potential for enhanced treatment effectiveness while minimizing adverse effects.

### 5.4. Radiation Therapy

Radiation therapy is a common treatment for early stage localized prostate cancer, targeting locally advanced cancers and reducing the risk of metastasis. Despite significant progress, radiation therapy’s limited therapeutic window results in many patients experiencing cancer recurrence. Increasing radiation doses can damage healthy tissues, causing adverse effects. Emerging techniques like proton and carbon ion therapy aim to target tumor cells more precisely while minimizing harm to nearby healthy cells [111]. Approximately 60–70% of patients experience tumor recurrence (10–40%) after radical prostatectomy, with side effects such as radiation proctitis being avoidable through accurate patient positioning and thorough setup verification [112].

#### 5.4.1. Lutetium-177

For the treatment of castration-resistant prostate cancer (CRPC) in patients who have progressed beyond androgen signaling inhibitors (ASIs) and have finished at least one cycle of taxane-based chemotherapy, lutetium-177 prostate-specific membrane antigen (PSMA)-617 has been approved by the FDA and EMA. Approval was based on favorable outcomes in the VISION trial (NCT03511664). Positive results before chemotherapy initiation may encourage earlier adoption of this therapy. However, resistance mechanisms to PSMA radioligand therapy (RLT) include non-uniform dose distribution, less controlled radiation effects on visceral organs, insufficient cytotoxic effects of 177Lu, and tumor mutational burden conferring resistance [67,113].

#### 5.4.2. Radium-223 Dichloride

Radium-223 dichloride (Xofigo) is a radiopharmaceutical approved by the FDA for managing bone pain in metastatic CRPC patients, as demonstrated in the ALSYMPCA study (2013). Radium-223 releases alpha particles that damage DNA strands and destroy cancer cells. It specifically targets bone metastases by mimicking calcium and forming compounds with hydroxyapatite, common in areas with significant bone remodeling [114,115]. The short-range and high linear energy transfer of alpha particles enable focused treatment, minimizing harm to healthy cells. Radium-223 reduces pathological bone turnover and irradiates tumors, prolonging overall survival in symptomatic patients with numerous bone metastases in CRPC [115,116].

### 5.5. Immunotherapy

Immunotherapies enhance the patient’s immune system to fight cancer cells. The FDA has approved three immunotherapies for CRPC: an immune-cell-based vaccine and two immune checkpoint inhibitors (ICI) [73]. Prostate cancer cells express proteins like PSA and prostatic acid phosphatase (PAP), potential targets for antigen-based vaccines [117].

#### 5.5.1. Sipuleucel-T

Sipuleucel-T is an FDA-approved cellular immunotherapy for men with metastatic castration-resistant prostate cancer (mCRPC) who are asymptomatic or have minimal symptoms. It targets prostatic acid phosphatase (PAP), an antigen present in most prostate cancer cells, by activating the immune system [118]. Approved on 29 April 2010, Sipuleucel-T showed an overall survival benefit for mCRPC patients. This treatment involves collecting autologous peripheral blood mononuclear cells, including antigen-presenting cells (APCs), from the patient, activating them with a recombinant fusion protein (PAP-GM-CSF), and reinfusing them into the patient over three treatments in six weeks [119]. PAP is an excellent candidate for prostate cancer vaccines because it is restricted to prostate tissue and seen in approximately 95% of prostate malignancies.

The specific mechanisms of Sipuleucel-T include the following: This enhances the immune response, correlating with improved overall survival (OS). Increased APC activation is seen with subsequent infusions, suggesting the initial infusion primes the immune system while boosting subsequent responses. Sipuleucel-T induces targeted immune responses against PAP and PA2024 [120]. Following the initial immune response, Sipuleucel-T broadens its response to other tumor antigens, stimulating immune responses against secondary antigens (such as E-RAS, KLK2, K-RAS, LGALS3, and LGALS8), linked to enhanced survival benefits [120,121]. Neoadjuvant Sipuleucel-T induces systemic antigen-specific immune responses [122]. Sipuleucel-T was the first FDA-approved vaccine for treating existing cancer, with trials showing minimal adverse events. The IMPACT trial reported that grade 3 or higher adverse events occurred in 31.7% of Sipuleucel-T patients and 35% of placebo patients, with common low-grade events being chills, fever, headache, muscle pain, and flu-like symptoms [118]. Subsequent trials have explored Sipuleucel-T for different prostate cancer stages and in combination with other treatments. In a phase II trial, Sipuleucel-T administered before prostatectomy increased tumor-infiltrating T cells, but there are no reports on improved pathological responses or delayed disease recurrence [122].

Phase III trials, D9901 and D9902A, showed that Sipuleucel-T provided a median overall survival benefit compared with placebo (23.2 months vs. 18.9 months; HR 1.50, *p* = 0.011) [119]. The IMPACT study, with overall survival as the primary endpoint, also demonstrated a significant survival benefit (25.8 months vs. 21.7 months; adjusted HR 0.78, *p* = 0.03) without differences in disease progression [118]. However, there are concerns about potential confounding due to differences in post-progression treatments [118]. Currently, Sipuleucel-T is recommended only for mCRPC patients who are asymptomatic or minimally symptomatic, have no liver metastases, have an ECOG performance status of 0–1, and have a life expectancy of more than six months [123]. It is not recommended for patients with neuroendocrine or small-cell histology [124].

#### 5.5.2. Dostarlimab

The FDA has approved dostarlimab and pembrolizumab, both monoclonal antibodies (mAbs) and immune checkpoint inhibitors (ICIs), for treating advanced solid tumors, including castration-resistant prostate cancer (CRPC). In 2017, pembrolizumab received approval for patients with metastatic solid tumors exhibiting mismatch-repair deficiency (dMMR) or high microsatellite instability (MSI-H) [125]. In 2020, the FDA expanded pembrolizumab’s indications to include patients with metastatic solid tumors having a high tumor mutational burden (TMB-H) [126]. This was the first tissue-agnostic approval, highlighting the potential of ICIs for various cancers.

**Figure 5:**
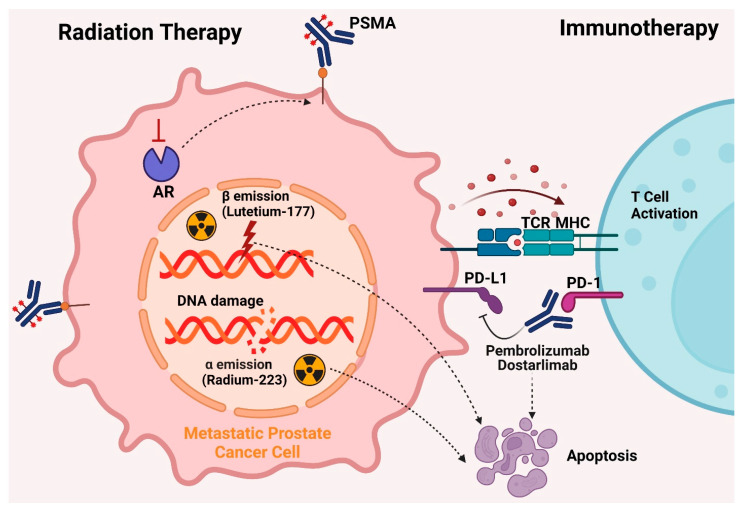
FDA-Approved Treatments for Castration-Resistant Prostate Cancer (CRPC). Radium-223 and Lutetium-177 are radiopharmaceuticals used in the treatment of CRPC. Radium-223 emits high-energy alpha particles, while Lutetium-177 emits beta particles. These particles directly damage the DNA of cancer cells, leading to cell death. Both treatments specifically target bone metastases, a common complication in CRPC, effectively reducing tumor burden. The precision of these therapies helps minimize damage to surrounding healthy tissues, providing a focused approach to treat metastatic bone lesions. Pembrolizumab, an immune checkpoint inhibitor, is a key immunotherapy approved for CRPC. It binds to PD-1 receptors on T cells, blocking the interaction between PD-1 on T cells and PD-L1 on tumor cells. This blockade lifts the immune suppression exerted by the tumor, thereby activating the immune system to recognize and attack cancer cells. The immune response is initiated by the detection of neoantigens presented on major histocompatibility complexes (MHC) by tumor cells. This mechanism enhances the body’s natural defense against cancer by overcoming one of the major immune evasion strategies employed by tumors.

In 2021, the FDA approved dostarlimab, another PD-1 inhibitor, for adult patients with dMMR/MSI-H recurrent or advanced solid tumors that had progressed despite previous treatments [127]. This approval was based on the GARNET trial, which demonstrated a 41.6% overall response rate (ORR) in patients treated with dostarlimab. Both dostarlimab and pembrolizumab function by inhibiting the programmed cell death-1 (PD-1) receptor, enhancing the immune system’s ability to target cancer cells [128]. Despite their efficacy in several cancers, ICIs have shown limited success in treating metastatic CRPC due to the immunosuppressive tumor microenvironment, particularly in patients with visceral metastases, prior chemotherapy, or stable microsatellite illness [129]. The interaction between PD-1 on immune cells and PD-L1/PD-L2 on cancer cells suppresses T-cell activation, leading to immune evasion by tumors [130]. Nivolumab and pembrolizumab have shown efficacy against various cancers, leading to regulatory approvals [131]. However, monotherapy with ICIs has yielded limited results in CRPC, with objective response rates (ORRs) of 5% in patients with PD-L1 CPS ≥1 and 3% in those with negative PD-L1 expression [129]. The limited efficacy is due to factors like T-cell anergy, low CD8+ T-cell infiltration, and the protective tumor microenvironment [132]. Studies have indicated that combining pembrolizumab with enzalutamide results in significant and durable responses in mCRPC, regardless of PD-L1 expression or DNA repair deficiencies [133]. Understanding immune evasion mechanisms in prostate cancer is essential for identifying effective immunotherapeutic targets and optimizing treatment timing. Dostarlimab and pembrolizumab represent promising advancements in the treatment of CRPC, offering new therapeutic options for patients with advanced disease. Future research should focus on combination therapies and further elucidating the mechanisms of immune evasion to improve treatment efficacy and patient outcomes. Figure 5 illustrates the FDA-approved treatments for managing CRPC, including current therapies like immunotherapy and radiation therapy.

### 5.6. PARP Inhibitors

**Figure 6:**
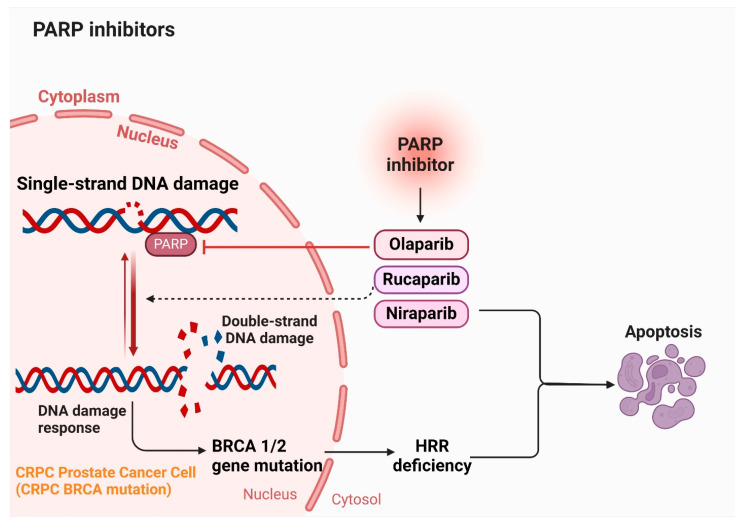
Mechanism of action of PARP inhibitors (Olaparib, Rucaparib, and Niraparib) in treating castration-resistant prostate cancer (CRPC). PARP inhibitors such as Olaparib, Rucaparib, and Niraparib block the PARP enzyme, which is crucial for repairing single-strand DNA breaks. Inhibition of PARP leads to the persistence of single-strand breaks, which during DNA replication, result in double-strand breaks. Cells with BRCA1 or BRCA2 mutations exhibit homologous recombination deficiency (HRD). These cells are unable to efficiently repair double-strand breaks through the homologous recombination pathway. The accumulation of unrepaired double-strand breaks in HRD cells triggers cell death, effectively targeting cancer cells harboring BRCA mutations. This targeted approach exploits the inherent weaknesses in the DNA repair mechanisms of CRPC cells, providing a potent and specific strategy to combat this challenging form of cancer.

Four PARP inhibitors approved by both the FDA and the European Medicines Agency (EMA) are leading the way in extended treatment approaches using targeted therapies for specific cases of prostate cancer. These drugs include olaparib (Lynparza), rucaparib (Rubraca), niraparib (Zejula), and talazoparib (Talzenna) [70,134,135]. They are indicated for patients with prostate cancer that has spread and is no longer responsive to hormone therapy, known as castration-resistant disease. Eligibility for these inhibitors requires specific genetic abnormalities that impair cells’ ability to repair DNA damage. In treating metastatic prostate cancer, conventional therapies inhibit hormones to halt their support for cancer growth and spread. However, PARP inhibitors function differently by targeting the PARP protein, crucial for repairing single-strand DNA breaks (SSBs) [136]. Each of these inhibitors varies in their specificity for different PARP proteins. Notably, rucaparib exhibits the least selectivity, binding not only to PARP1 but also to other PARP proteins and additional targets such as hexose-6-phosphate dehydrogenase, leading to diverse effects. Despite their promise, limitations exist in current treatments, underscoring the need for alternative therapies or natural compounds. Each treatment modality carries specific limitations and side effects, advocating for a combination approach to enhance clinical outcomes while minimizing adverse effects. Ongoing research aims to develop new compounds and therapies that can overcome these limitations, offering more effective and safer options for patients with castration-resistant prostate cancer (CRPC). Figure 6 illustrates the role of PARP inhibitors in the management of CRPC.

## 6. Role of Natural Compounds in Regulating AR-V7 in Prostate Cancer

Medicinal plants have long been valuable sources of therapeutic agents, providing a wealth of secondary metabolites with extensive structural diversity and a broad range of biological activities. In prostate cancer, AR-V7 is a critical driver of castration resistance, contributing to tumor invasion, metastasis, resistance to hormone therapy, and biochemical recurrence [137]. Table 3 illustrates the promising role of natural compounds in the treatment of castration-resistant prostate cancer (CRPC), specifically in the regulation of AR-V7.

## 7. The Effectiveness and Mechanism of Action of the Natural Compounds in Castration-Resistant Prostate Cancer

Natural compounds extracted from medicinal plants have demonstrated substantial potential in addressing castration-resistant prostate cancer (CRPC). These phytochemicals exhibit diverse biological activities, including the inhibition of cancer cell proliferation, the induction of apoptosis, and the modulation of critical signaling pathways integral to prostate cancer pathogenesis. Notably, they have the capability to regulate the androgen receptor splice variant 7 (AR-V7), a pivotal factor implicated in therapeutic resistance and the advancement of CRPC. By specifically targeting AR-V7 and its associated molecular pathways, these natural compounds not only exhibit intrinsic therapeutic properties but also synergistically augment the efficacy of existing therapeutic modalities. This dual approach enhances the overall therapeutic landscape, presenting a promising avenue for the development of novel, integrative strategies in prostate cancer management.

### 7.1. Berberine in Cancer Therapy

Researchers are increasingly focusing on alternative medicines with low toxicity and fewer side effects for preventing and treating prostate cancer. Natural products, including berberine, have shown significant promise as chemotherapeutic agents [156]. Berberine exhibits a wide range of pharmacological activities, such as antimicrobial, anti-inflammatory, and anti-diabetic properties. Recent studies highlight its extensive antitumor activity, including inhibiting cancer cell proliferation, inducing apoptosis, and preventing invasion and migration [156,157]. Berberine specifically decreases the migration and invasion abilities of highly metastatic prostate cancer cells. It downregulates several mesenchymal genes involved in the epithelial–mesenchymal transition (EMT), which is critical for cancer metastasis [158,159]. Notably, higher expressions of BMP7, NODAL, and Snail genes correlate with poorer survival rates in metastatic prostate cancer patients, making them potential therapeutic targets [159]. Additionally, berberine enhances the sensitivity of resistant cancers to chemotherapy and radiotherapy by inhibiting HIF-1α and VEGF expressions, thus increasing radiosensitivity [160]. It also triggers tumor cell apoptosis through the p53-mediated intrinsic apoptotic pathway and elevates reactive oxygen species (ROS) production [161].

In androgen-dependent and castration-resistant prostate cancer (CRPC) cells, berberine suppresses AR transcriptional activity and the expression of AR-regulated genes. It promotes the proteasome-mediated degradation of AR proteins, inhibits the interaction between AR and heat shock protein 90 (Hsp90), and prevents AR nuclear translocation. This results in the degradation of both full-length AR and AR splice variants lacking the ligand-binding domain (ARΔLBDs) [138]. These findings underscore berberine’s potential as a promising treatment for CRPC, warranting further research and development to target AR signaling pathways effectively.

### 7.2. Curcumin in Cancer Therapy

Curcumin, a bioactive compound from turmeric, exhibits significant anti-cancer properties. It inhibits cancer cell growth and survival, reduces inflammation, prevents invasion, and induces apoptosis in malignant cells [162]. Studies have shown curcumin’s effectiveness against various disorders due to its anti-inflammatory, antioxidant, and antimicrobial properties [163,164].

In prostate cancer (PCa), curcumin interacts with multiple molecular targets, such as mTOR, p53, Ras, PI3K/Akt, and Wnt-β catenin, disrupting cancer growth at various stages. It demonstrates chemopreventive attributes, capable of stopping, reversing, and inhibiting cancer development [165,166]. Animal experiments have shown curcumin’s dose-dependent chemopreventive benefits against PCa [167]. Moreover, curcumin acts as a chemo- and radiosensitizer, enhancing the efficacy of conventional therapies [168].

Curcumin’s safety profile is well-established [169], with clinical research validating its low toxicity and minimal side effects at all dosages [170]. The U.S. Food and Drug Administration (USFDA) has categorized curcumin as Generally Recognized As Safe (GRAS), recommending a daily intake of 8 to 12 g [171]. Curcumin’s anti-tumor properties extend to various cancer types, including prostate cancer [172,173,174]. It inhibits the overexpression of oncogenes and signaling pathways involved in PCa, such as Bcl-2 [175], androgen receptor (AR) signaling, EGFR, HER2, Cyclin D1, COX-2, MMPs, Akt, NF-κB, AP-1, STAT3, MAPK, JAK/STAT, and PI3K/Akt/mTOR. Curcumin has shown efficacy similar to traditional chemotherapy medications in inhibiting the proliferation of androgen-dependent (LNCaP) and androgen-independent (PC-3) prostate cancer cells [176]. These findings underscore curcumin’s potential as a promising treatment for prostate cancer, warranting further research and development to fully harness its therapeutic benefits [177].

### 7.3. Cryptotanshinone (CTS)

Cryptotanshinone (CTS), a diterpene quinone from Danshen (Salvia miltiorrhiza Bunge), has shown promise in cancer treatment due to its specific cytotoxicity towards prostate cancer cells, with minimal effects on normal cells. It induces apoptosis and cell cycle arrest in cancer cells, evidenced by upregulating cyclin A1, cyclin B1, and Cdc25c, leading to G2/M phase arrest. Additionally, CTS enhances the sensitivity of drug-resistant prostate cancer cells to apoptosis mediated by Fas and various chemotherapeutic agents, including doxorubicin and cisplatin [178]. CTS is known for its selective inhibition of the STAT3 signaling pathway, without affecting other STAT family proteins. It inhibits the transcriptional activity of the activated androgen receptor (AR) in prostate cancer cells without altering AR mRNA or protein levels. This inhibition is mediated through the suppression of AR target genes, such as NKX3.1, TMPRSS2, and PSA. Furthermore, CTS interferes with the interaction between AR and the histone demethylase LSD1, preventing the demethylation of histone H3 at lysine 9 (H3-K9), thus inhibiting AR-mediated gene activation and prostate cancer growth [179]. Recent studies suggest that the AR-LSD1 interaction is a primary target for CTS-mediated inhibition, highlighting the potential of CTS as a novel therapeutic agent in the treatment of castration-resistant prostate cancer (CRPC). The effects of CTS on the assembly of the AR, JMJD2C, and LSD1 complex on chromatin warrant further investigation to fully understand its therapeutic potential and mechanisms of action [180].

### 7.4. Epigallocatechin-3-Gallate (EGCG)

Epigallocatechin-3-gallate (EGCG), the primary catechin in green tea, has been extensively studied for its potential anti-cancer properties. Research has demonstrated that EGCG can significantly reduce prostate cancer cell proliferation [181]. Various mechanistic studies have shown that EGCG selectively induces apoptosis and inhibits the proliferation of several cancer cell lines, including DU145, PC-3, and LNCaP, in a dose-dependent manner [182].

Chronic inflammation is a known contributor to tumor development, and EGCG has been shown to modulate inflammatory mediators such as COXs, MMPs, IL-1β, and TNF-α. These inflammatory markers, when overexpressed, often correlate with poorer cancer prognoses. EGCG effectively inhibits these mediators, thus reducing inflammation and potentially curbing cancer progression [183].

In prostate cancer cells, EGCG has demonstrated antiproliferative effects regardless of androgen sensitivity. It induces apoptosis and disrupts the cell cycle, causing a dose-dependent arrest in the G0/G1 phase in both androgen-sensitive and insensitive cells. This arrest is mediated by the induction of cyclin kinase inhibitor WAF1/p21 and an increase in p53 levels in LNCaP cells but not in DU145 cells, which carry mutant p53. This indicates that EGCG can negatively regulate prostate cancer cell growth by affecting mitogenesis and inducing apoptosis in a cell-type-specific manner [184]. Furthermore, EGCG’s regulatory functions extend to intracellular calcium mobilization, crucial for cancer development. High doses of EGCG cause an initial rise in intracellular calcium, interacting directly with plasma membrane proteins and phospholipids. This action is critical in various cell types, including smooth muscle cells and mast cells, where EGCG has been shown to activate calcium influx through different channels [185]. Overall, EGCG’s multifaceted mechanisms of action, including anti-inflammatory effects, cell cycle arrest, and apoptosis induction, highlight its potential as a therapeutic agent in prostate cancer treatment. Further studies and clinical trials are essential to fully understand its efficacy and therapeutic application in cancer management.

### 7.5. Fisetin

Fisetin, a dietary tetra-hydroxyflavone found in various fruits and vegetables, has shown potential as a chemotherapeutic agent against prostate cancer. Studies reveal that fisetin inhibits several cancer-causing pathways in both in vitro and in vivo models [186]. It demonstrates anti-cancer properties by stabilizing microtubules, reducing cancer cell motility, invasion, and proliferation. In prostate LNCaP cells, fisetin induces G1 phase cell cycle arrest by downregulating cyclins and cyclin-dependent kinases and activates apoptotic pathways [187]. Research on prostate cancer cell lines (LNCaP, DU145, PC-3) has demonstrated fisetin’s effectiveness in reducing tumor growth by competing with AR ligands, lowering AR stability, and enhancing TRAIL-induced apoptosis [188]. Additionally, fisetin decreases the activities of MMP-2 and MMP-9, which are essential for cancer cell invasion and migration [189]. This inhibition occurs through the suppression of the JNK and PI3K/Akt signaling pathways, reducing metastasis potential [188].

Fisetin’s anti-metastatic effects are further supported by its ability to inhibit NF-κB and activator protein 1 (AP1), preventing their nuclear translocation and subsequent binding to the regulatory sequences of MMP-2 and MMP-9 [190]. This action disrupts the extracellular matrix (ECM) breakdown necessary for cancer cell migration. Furthermore, fisetin induces cell cycle arrest in the G1 phase by decreasing CDK2, CDK4, CDK6 activities, and the levels of cyclins D1, D2, and E [188].

At the molecular level, fisetin targets microtubules by binding to β-tubulins, preventing cell proliferation, migration, and invasion, while increasing α-tubulin acetylation. This stabilization of microtubules disrupts mitosis and cell growth in prostate cancer cells [187]. Fisetin also influences Y-box binding protein-1 (YB-1), which regulates EGFR expression and epithelial-to-mesenchymal transition (EMT). By binding to the cold shock domain (CSD) of YB-1, fisetin suppresses YB-1 phosphorylation and AKT, inhibiting EMT [191]. Fisetin demonstrates significant anti-cancer potential by modulating key molecular pathways, inducing apoptosis, and inhibiting cell proliferation and metastasis in prostate cancer cells. Its ability to target multiple pathways highlights its promise as a therapeutic agent in prostate cancer treatment [192].

### 7.6. Genistein

Genistein, a prominent constituent of soy isoflavones, has demonstrated significant inhibitory effects on CRPC as evidenced by numerous studies. Genistein exerts a broad range of molecular effects, including inflammation inhibition, apoptosis promotion, and regulation of steroid hormone receptors and metabolic pathways [193]. Moreover, androgen deprivation therapy (ADT) may upregulate the PI3K/AKT pathway due to inhibitory feedback loops within the androgen receptor (AR) signaling pathway, and upregulation can occur independently of AR-V7, potentially leading to increased AKT1 expression without direct regulation by AR-FL [194]. The comprehensive understanding of genistein’s molecular mechanisms and its integration with current therapeutic strategies underscores its potential as a valuable agent in treating CRPC. The usage of a particular dosage, the physiological condition of the test organism, and food are likely to have an impact on the effectiveness of genistein in the context of cancer therapy. It is still unclear how and to whom genistein should be given to produce the intended health effects, and how it works at the beginning and end stages of the cancer process. In light of the possibility of cancer, it is also critical to respond to the question of whether genistein-containing supplementation is safe for women. The explanation for how genistein in its nano and micro form could function is another crucial factor. These days, research on phenolic compounds in their nanoscale forms has gained significant importance. Their bioavailability varies in the nano form. Absorption, systemic circulation penetration, and cellular usage are the three fundamental stages that characterize bioavailability. In comparison to macromolecules, the reduction of materials at the nanoscale can result in the creation of novel physical, chemical, and biological properties. It is important to highlight that the literature on the topic being discussed still lacks study [195]. However, the amounts of calcitriol in prostate tissues may be reduced by genistein. In several forms of prostate cancer, androgen deprivation treatment is widely recognized. In order to halt the prostate’s growth, this treatment lowers testosterone levels. Genistein’s capacity to cause apoptosis, suppress important components of cell growth, and alter miRNA expression is proven. Its therapeutic effectiveness has been further boosted by conjugating it with gold nanoparticles and incorporating it into specific liposomes, therefore providing a more selective therapy method. Furthermore, genistein’s extensive significance in managing prostate cancer is highlighted by its potential benefits in reducing the negative effects of hormonal therapy [196]. Prospective research on the use of genistein as an antimetastatic drug was prompted by epidemiologic studies that suggested genistein may have antimetastatic efficacy against PCa in people. Significant proof that genistein can alter the potential for human PCa to spread has been obtained from these investigations. Genistein therapy reduced metastatic load and decreased cell detachment in an orthotopic human PCa model, while leaving the primary tumor size unchanged [197].

### 7.7. Garcinia Mangostana/α-Mangosteen

Mangosteen (*Garcinia mangostana* L.) contains xanthones, bioactive substances with significant anticancer properties. Among them, α-mangostin has shown potential due to its ability to inhibit cell proliferation, induce apoptosis, and impede metastasis in prostate cancer cells [198]. Recent studies highlight its effectiveness in promoting androgen receptor (AR) degradation and activating the unfolded protein response pathway, crucial for targeting CRPC (Castration-Resistant Prostate Cancer) [199]. Researchers have found that α-mangostin significantly reduces the viability of prostate cancer cells (PC3 and 22Rv1) by inducing cell cycle arrest through CDK4 inhibition and apoptosis via caspase-3 activation [200]. In vivo studies using an athymic nude mouse model confirmed α-mangostin’s capability to degrade both AR and AR-V7, demonstrating substantial efficacy in reducing cancer growth, especially in CRPC-like conditions. Notably, α-mangostin was the only treatment to show considerable reductions in tumor development, surpassing traditional treatments like bicalutamide. Further investigations revealed that α-mangostin treatment led to decreased expression of genes regulated by AR and AR-V7 in tumor tissues. Despite some discrepancies in AR and AR-V7 expression outcomes between in vitro and in vivo studies, α-mangostin’s promising results warrant more research to fully understand its mechanisms and therapeutic potential against CRPC. These findings position α-mangostin as a valuable candidate for developing new treatments for prostate cancer, particularly in overcoming resistance to conventional therapies [201].

### 7.8. Ginsenosides

The main pharmacologically active ingredients in ginseng are ginsenosides. These compounds are the primary bioactive constituents responsible for ginseng’s medicinal properties, particularly ginsenoside 20(S)-protopanaxadiol-aglycone. This specific ginsenoside has been demonstrated to suppress androgen receptor (AR) expression and inhibit AR transactivation even under androgen-independent conditions. Research has shown that ginsenoside 20(S)-protopanaxadiol-aglycone effectively inhibits the growth of castration-resistant prostate cancers (CRPC) that express both full-length AR (AR-FL) and AR splice variants (AR-Vs). Additionally, this ginsenoside has been found to either prevent or decelerate the progression of CRPC following androgen deprivation therapy in xenograft models [202]. These findings highlight the potential of ginsenoside 20(S)-protopanaxadiol-aglycone as a therapeutic agent for managing CRPC by targeting both AR-FL and AR-Vs. This dual targeting capability is particularly important, given the role of AR-Vs in promoting treatment resistance and disease progression in CRPC.

### 7.9. Honokiol

Honokiol, a natural compound extracted from the magnolia tree’s seed cones and bark, has shown significant anticancer properties [203]. In preclinical models and in vitro studies, honokiol has demonstrated its ability to induce apoptosis in cancer cells through multiple signaling pathways, including NF-κB, STAT3, EGFR, and mTOR. It has also displayed favorable pharmacokinetics and bioavailability in animal models, making it a promising candidate for clinical studies [204]. Honokiol targets several molecular pathways involved in cancer progression. It binds effectively to apoptotic factors, chemokines, transcription factors, and kinases, enhancing its therapeutic potential. In the human prostate, honokiol impacts cell cycle regulation and contraction [205]. It has been shown to induce smooth muscle relaxation and inhibit prostate growth, offering potential treatment for benign prostatic hyperplasia [206].

Studies on honokiol’s effects on prostate cancer cells have revealed that it induces apoptotic DNA fragmentation regardless of androgen responsiveness or p53 status [207]. In mouse models, honokiol inhibits the growth of PC-3 xenografts without causing significant side effects. When combined with docetaxel, honokiol synergistically suppresses cell survival and reduces tumor areas in the C4-2 xenograft model [208].

Honokiol treatment reduces AR protein levels in both androgen-responsive and androgen-independent prostate cancer cells through proteasomal degradation and decreased AR mRNA levels [147]. By targeting AR signaling pathways, honokiol impairs AR-mediated transcriptional activity, enhancing the efficacy of existing prostate cancer therapies and offering potential applications for treating castration-resistant prostate cancer (CRPC).

### 7.10. Luteolin

The flavonoid luteolin (3′,4′,5,7-tetrahydroxy flavone), known for its anti-inflammatory and antioxidative properties, is naturally found in perilla seeds, celery, green pepper, and parsley. Recent studies have highlighted luteolin’s potential as a therapeutic agent in the management of castration-resistant prostate cancer (CRPC) using a rat model. The anti-CRPC properties of luteolin are primarily attributed to its suppressive effect on AR-V7. In dietary interventions, luteolin has demonstrated efficacy in preventing the onset of early prostate carcinogenesis in TRAP rats without adverse side effects. Additionally, it has been shown to inhibit tumor growth in CRPC by inducing apoptosis. A significant mechanism underlying these effects is the upregulation of miR-8080, which is induced by luteolin supplementation. This miRNA plays a pivotal role in decreasing AR-V7 protein levels, thereby inhibiting tumorigenesis and mitigating resistance to enzalutamide, a commonly used anti-androgen therapy in CRPC [148]. These findings underscore luteolin’s potential to enhance the effectiveness of existing CRPC therapies and its promise as a novel therapeutic strategy targeting AR-V7 and associated pathways, thereby improving treatment outcomes for patients with advanced prostate cancer.

### 7.11. Quercetin

Quercetin, a flavonoid found in fruits and vegetables, possesses anti-inflammatory, antioxidant, and anticancer properties. It disrupts cancer cell cycles and induces apoptosis. Research has demonstrated quercetin’s therapeutic benefits in various cancers, including prostate cancer. It inhibits androgen receptor (AR) and prostate-specific antigen (PSA) activity, crucial in prostate cancer etiology [209]. Quercetin modulates signaling pathways like PI3K/Akt/mTOR, Wnt/β-catenin, and MAPK/ERK1, promoting apoptosis and autophagy, and reducing metastasis by lowering VEGF and MMP release [210]. Quercetin’s combination with chemotherapeutic drugs, such as docetaxel, shows enhanced efficacy in reducing cell viability and metastasis. Studies reveal that quercetin combined with docetaxel results in significant apoptosis and PI3K/Akt pathway inhibition. Co-administration of quercetin with docetaxel has shown promising results in docetaxel-resistant xenograft tumor models, indicating potential for more effective and economical treatments with fewer side effects [211]. Further research is needed to fully understand quercetin’s role in enhancing chemotherapy and reducing drug resistance in prostate cancer [212].

### 7.12. Resveratrol

Resveratrol, a naturally occurring stilbenoid, demonstrates significant anti-cancer properties with minimal side effects [213]. It effectively targets the tumor microenvironment (TME) and androgen receptor (AR) in prostate cancer [214], inducing growth inhibition, cell cycle arrest, apoptosis, and metastasis suppression [215]. Resveratrol generates reactive oxygen species (ROS) [216], causing oxidative stress and cellular damage, leading to cancer cell apoptosis through multiple signaling pathways [217]. However, its poor solubility, low bioavailability, and rapid metabolism limit its efficacy. Utilizing nanoparticles as a delivery system can enhance resveratrol’s stability, solubility, and therapeutic effectiveness in prostate cancer cells. This enhanced delivery system ensures more effective drug performance, addressing the common limitations faced by traditional administration methods and making resveratrol a promising candidate for advanced prostate cancer treatments.

### 7.13. Silibinin

Silibinin, a flavolignan extracted from the fruits of *Silybum marianum* (milk thistle), is increasingly recognized as a promising candidate for cancer prevention and treatment. Silibinin exerts its anti-cancer effects by impeding cell proliferation through targeting the androgen receptor (AR). Notably, silibinin has been shown to decrease the expression of prostate-specific antigen (PSA), an AR target gene, particularly under androgen-stimulated conditions. This reduction in PSA expression highlights silibinin’s potential role in disrupting AR signaling pathways, making it a viable therapeutic agent for managing prostate cancer, including castration-resistant prostate cancer (CRPC) [218].

### 7.14. Sulforaphane

Sulforaphane (SFN), a natural compound abundantly found in broccoli sprouts and various cruciferous vegetables, demonstrates potent inhibitory effects on the expression of both full-length androgen receptor (AR-FL) and the variant AR-V7 [70,99]. Moreover, research indicates that SFN enhances the anticancer efficacy of anti-androgens by exerting its suppressive action on AR [152,219]. The intricate interplay between AR and other signaling pathways in PCa significantly modulates AR’s transactivation capacity, thereby contributing to early development of CRPC [220]. The dysregulated expression of AR, in conjunction with other signaling pathways supporting and propagating PCa cells, leads to the upregulation of target genes pivotal for cell survival, proliferation, secretion, and lipid synthesis. These genes encompass a wide array of transcription factors, cell cycle regulators, and proteins crucial for cancer progression. Thus, investigating the crosstalk between AR and key signaling pathways in PCa emerges as a crucial strategy to impede the progression of PCa and hinder its transformation into CRPC [221].

### 7.15. Triptolide

Triptolide (TPL), derived from the Chinese herb Tripterygium wilfordii, is recognized as a potent cancer-preventive substance. It exerts its effects by inhibiting XPB/CDK7-mediated phosphorylation of both full-length androgen receptor (AR-FL) and the variant AR-V7 at Ser515. Notably, TPL demonstrates a significant inhibitory effect on the binding of androgen-induced AR to the androgen response element (ARE) located in the enhancer region of the PSA/KLK3 gene. This mechanism underscores the potential of TPL as a therapeutic agent for targeting AR-driven pathways in prostate cancer [154].

## 8. Pro-Oxidative Activity of Natural Compound in Cancer Treatment

Numerous studies have shown that phenolic compounds found in food and medicinal plants possess chemopreventive and anticancer effects. This dual action is attributed to their unique capacity to influence the redox state of cells. These compounds act as pro-oxidants to selectively kill cancer cells while also stimulating an antioxidant response to prevent cancer development. Antioxidant polyphenols can scavenge reactive oxygen species (ROS), chelate metals that induce oxidative stress, and/or activate ARE-controlled genes encoding detoxifying enzymes. These mechanisms collectively mitigate oxidative stress-mediated cellular damage, which is associated with a reduced risk of cancer [222]. Phenolic compounds exhibit this duality by behaving as both antioxidants and pro-oxidants, depending on the cellular context. As antioxidants, they reduce oxidative stress by neutralizing ROS, enhancing cellular defense mechanisms. As pro-oxidants, they increase ROS levels selectively in cancer cells, inducing cytotoxicity. This ability to modulate ROS levels makes them potent agents in cancer therapy, where balancing oxidative stress can lead to the targeted destruction of cancer cells while protecting healthy cells. By harnessing their ability to manipulate the redox state within cells, these compounds can selectively target cancer cells for destruction while simultaneously offering protective antioxidant effects, making them valuable adjuncts in the fight against cancer.

### 8.1. Cytotoxic Effects of Plant Polyphenol Compounds in Cancer Treatment

Research indicates that flavonoids, a significant group of plant polyphenols, exhibit cytotoxic effects primarily at high micromolar concentrations. Factors influencing plasma levels of dietary flavonoids include functional categories and daily consumption. However, achieving sufficient plasma concentrations through oral dosing to elicit antiproliferative and cytotoxic effects remains challenging [222]. Plant-based antioxidants such as flavonoids, phenolic acids, tannins, and phenolic diterpenes are central to this action. Interestingly, flavonoids like quercetin can convert into cytotoxic prooxidant metabolites in vitro, highlighting their dual role [223]. Therefore, while flavonoids constitute two-thirds of dietary phenolics, their therapeutic efficacy relies on high intake levels, which are difficult to achieve through oral consumption alone [223].

### 8.2. The Limitations and Challenges of Using Natural Compounds in Treating CRPC

Plant extracts and their various constituents have garnered attention for their therapeutic potential, particularly in reducing cancer-related mortality. Despite their ancient use, it was not initially evident that these natural substances could be effective. Researchers have identified numerous herbal compounds with anticancer properties, but several challenges remain. These include broad-spectrum activity, toxicity, water insolubility, passive targeting, and herb-drug interactions. For instance, clinical trials with pomegranate juice and extract over six months did not significantly reduce plasma PSA levels due to the low serum dose and the time-consuming nature of these studies, which often take months to years to yield results [224].

To achieve successful therapeutic outcomes, several constraints with natural substances must be addressed. A significant limitation of many natural compounds is their limited bioavailability, making it challenging to reach effective in vivo concentrations through oral intake. Long-term toxicity of these phytochemicals needs further investigation due to the high amounts ingested during treatment. Comprehensive molecular target profiling is also necessary to alleviate concerns about the potential adverse effects of their multi-targeting efforts. Clinical trial data on phytochemicals are limited compared to numerous pre-clinical studies, indicating the need for more clinical research at every stage.

Additionally, discovering new phytochemicals with improved bioavailability and anti-cancer potential should be a priority. Despite these obstacles, natural products hold promise as supplementary or alternative cancer treatments. Elucidating precise mechanisms of action based on chemical structure will aid in discovering new natural compounds with notable anti-cancer properties, beneficial for drug development and combination optimization. This can lead to less toxic or non-toxic treatments for prostate cancer, offering substantial health benefits [143]. Most research focuses solely on drug efficacy, often overlooking potential side effects, and some studies have yet to fully clarify the precise molecular mechanisms of active ingredients in herbal remedies for prostate cancer therapies. Therefore, research on medication safety and network pharmacology analysis techniques should be integrated into studies on plant medicine molecules. Detailed experimental verification and extensive drug target protein screening can lead to a better understanding of their mechanisms of action [225].

### 8.3. Limitation of Castration-Resistant Prostate Cancer Treatment

As prostate cancer progresses to the castration-resistant stage, therapeutic options become increasingly limited compared to early stages [226]. While chemotherapy, newer hormonal treatments, immunotherapy, and targeted medicines can provide temporary disease control, they are not curative. Eventually, resistance to these treatments may develop as well. Prostate cancer cells may become resistant to hormone therapy after initially responding to Androgen Deprivation Therapy (ADT), leading to the emergence of CRPC [227]. The reasons behind this resistance are intricate and not completely comprehended, posing challenges for effective intervention [227]. Castration-resistant prostate cancer is a heterogeneous disease characterized by variations in biology, genetic alterations, and therapy response across patients [228]. The diversity in CRPC patients presents a challenge in developing targeted medicines that are effective for all individuals [229]. Therapies for CRPC, such as chemotherapy, targeted therapy, and immunotherapy, may result in notable adverse effects such as fatigue, nausea, diarrhea, alopecia, immunosuppression, and neuropathy [230]. Balancing these adverse effects with therapy effectiveness is crucial for optimizing patient results.

New treatments for CRPC, such as targeted medicines and immunotherapies, may come with high costs, which may hinder some patients from accessing them, especially in areas with scarce healthcare resources or insufficient insurance coverage. The prognosis for people with CRPC remains unfavorable, particularly when the cancer has metastasized to other organs, despite therapy improvements. Newer treatments show better overall survival and progression-free survival than traditional therapies, although the extent of improvement varies among individuals, and long-term results are still rather limited. Managing CRPC requires carefully weighing the potential therapeutic benefits against its effects on quality of life. Some therapies may extend lifespan but have notable adverse reactions that can impact physical capabilities, emotional health, and overall well-being. Collaborative decision-making between patients and healthcare providers is essential in taking these aspects into account when selecting treatment alternatives. Prostate cancer is a complex disease that poses numerous challenges in its diagnosis and treatment. Despite advancements in medical science, the management of prostate cancer often involves navigating through a range of treatment options, each with its own set of limitations and potential side effects. Surgical interventions such as radical prostatectomy and radiation therapy aim to remove or destroy cancerous cells, but they can lead to complications such as urinary incontinence and erectile dysfunction. Hormone therapy, which suppresses the production of testosterone to slow cancer growth, may result in side effects like hot flashes and loss of libido. Moreover, the emergence of CRPC, where the cancer progresses despite hormonal therapy, further complicates treatment. While newer therapies such as chemotherapy, targeted therapy, and immunotherapy have shown promise in extending survival, they also bring their challenges, including toxicity and resistance development.

## 9. Recommendations, Challenges, Future Perspectives, and Concluding Remarks

Various strategies have been explored to mitigate the lethality of mCRPC. The pursuit of enhanced treatment approaches continues, encompassing the combination of existing therapies, integration with novel therapeutic agents, and the application of precision medicine tailored to specific genetic abnormalities, such as the expression of AR-V7. Despite some advancements in prolonging the lifespan of mCRPC patients, the combinations tested so far have not demonstrated sufficient safety and effectiveness to be considered viable options for further enhancing outcomes in mCRPC. Since the recurrence of the disease is widespread throughout the body, treatment options are restricted to systemic therapies. Significant strides have been achieved in identifying, testing, and refining the molecular structures of these natural compounds [231]. Thus, it is recommended that the immediate next phase in the management and treatment of PCa involves transforming CRPC into a chronic condition using natural compound or combination therapy, thereby reducing the mortality rate related to this cancer type. Targeted therapies have revolutionized cancer treatments, providing opportunities for personalized and improved approaches. However, despite these advancements, challenges such as resistance mechanisms, identifying biomarkers, and determining optimal combination strategies still need to be addressed to enhance the efficacy and clinical impact of targeted drugs. Hence, it is imperative to comprehend the mechanisms of resistance and devise strategies to promptly overcome them. Additionally, it is crucial to acknowledge that some individuals may exhibit exceptional sensitivity to targeted therapies, leading to specific and severe adverse effects. Sustained research efforts and collaboration among academic institutions, industries, and regulatory authorities will play a pivotal role in advancing the realm of targeted therapies, ultimately contributing to enhanced outcomes for cancer patients.

## 10. Conclusions

To sum up, prostate cancer presents a serious threat to world health due to its rising prevalence and high death rates. Patients now have fresh hope thanks to advances in diagnostic and etiology research, as well as the development of innovative therapies that have significant effects on both people and communities. For physicians, castration-resistant prostate cancer poses a serious dilemma. However, new and potential therapy approaches have been uncovered by recent scientific advancements. Some of these novel therapeutic alternatives have received FDA clearance as a result of their better clinical trial results. Further investigation in this area might lead to significant advancements in cancer prevention, treatment, and diagnosis methods. Consequently, these results signify a major advancement in the battle against this cancer.

## Figures and Tables

**Table 1:** A list of FDA-approved drugs for the treatment of metastatic castration-resistant prostate cancer.

Drug (Brand) Name	Initial Approval Date by US-FDA	Mechanism of Action	Mode of Administration	Reference
Abiraterone Acetate (Zytiga)	28 April 2011	↓ 17α-hydroxylase CYP17 enzyme, testosterone, adrenal androgen, DHEA, androstenedione, DHT, PI3-kinase/Akt, Bcl-2, and c-Met, AR nuclear translocation, AR-DNA binding domain, PSA, and AR-V7	Oral	[58,59,60]
Apalutamide (Erleada)	14 February 2018	↓ AR at the ligand binding domain, AR nuclear translocation, DNA binding, AR-mediated transcription, co-factor recruitment, AR-V7	Oral	[61]
Cabazitaxel (Jevtana)	17 June 2010	↓ microtubular depolymerization, AR-V7, PSA, AR, Foxo-1, MCAK, and HSET	IV	[3,4,62,63]
Docetaxel (Taxotere)	19 May 2004	↑ Bcl-2, nuclear AR ↓ microtubular depolymerization, Bcl-2, Bcl-xL, TMPRSS2-ERG, AR-V7, PSA	IV	[5,62,64]
Darolutamide (Nubeqa)	30 July 2019	↓ AR-mediated transcription, AR nuclear translocation, F877L, 7878A, T878G levels	Oral	[6,65]
Dostarlimab	22 April 2021	↓ PD-1 receptor, PD-L1 and PD-L2 ligand		[7,8]
Enzalutamide (Xtandi)	31 August 2012	↓ AR nuclear translocation, AR-DNA binding, full-length AR translocation, co-factor recruitment, AR-V7, PSA	Oral	[9,51]
Leuprolide acetate (Lupron Depot)	1985	↓ LH, FSH, GnRH receptors, androgen synthesis	IV	[66]
Lutetium Lu-177 Vipivotide tetraxetan (Pluvicto)	23 March 2022	↓ PSMA, PSA	IV	[67,68]
Mitoxantrone (Novantrone)	1996	↓ topoisomerase II inhibitor, DNA replication	IV	[1]
Niraparib (Zejula)	27 March 2017	↑ PARP-DNA complexes ↓PARP	Oral	[69]
Olaparib (Lynparza)	19 December 2014	↓ PARP and PARP-DNA binding, ADT, HR, PTEN, TP53	Oral	[70,71]
Pembrolizumab	4 September 2014	↓ PD-1 receptor, PD-L1, PD-L2 ligand		[72,73]
Radium-223	2013	↓ PSA	IV	[1,74]
Rucaparib (Rubraca)	19 December 2016	↓ BRCA2, ATM, CHEK2, and BRCA1	Oral	[75]
Sipuleucel-T	April 2010	↑ antigen-presenting cells (APCs)	IV	[76]
Talazoparib (Talzenna)	16 October 2018	↓ PARP	Oral	[77]

↑, upregulated; ↓, downregulated.

**Table 2:** A classification of chemotherapeutic drugs used in the treatment of prostate cancer.

Types of Drugs	Name as Example	Mechanism of Action	Reference
Alkylating agents	Mechlorethamine melphalan	↑ nucleotide mismatching, ↑ DNA fragmentation	[78]
Antimetabolites	Decitabine	↑ Inhibit DNA methyltransferase, ↑ DNA hypomethylation, ↑ S Phase of cells	[79]
Topoisomerase (ii) inhibitors	Etoposide	↑ Inhibit both α and β isoforms of topoisomerase II	[80]
Mitotic inhibitors	Docetaxel	↓Mitosis, ↑ apoptosis ↓ Bcl-2	[81]
Mitotic Inhibitors (Vinca Alkaloids)	Vinorelbine	↓ Mitosis, ↓ Bcl-2, ↑ BAX [5]	[82]
Corticosteroids	Prednisone	↓ gene expression, ↓ Inflammatory transcription	[83]
Synthetic Analog	Ixabepilone	↑ Microtubule stability	[84]

↑, upregulated; ↓, downregulated.

**Table 3:** A comprehensive list of natural compounds studied for their potentiality in regulating androgen receptor (AR) and AR splice variant 7 (AR-V7).

Scientific Name/Chemical Compound	Plant Source	Plant Part	Efficacy	Mechanism	References
Berberine			Induction of apoptosis, suppression of cell proliferation, CRPC xenografts, and AR splice variant	↓ AR, AR translocation, AR-fL, ARΔLBDs, and HSP90	[138,139]
Curcumin/*Curcuma longa*			Inhibition of cell proliferation, programmed cell death, G2/M cell cycle arrest, and inflammation	↓ AR, PSA, ARE, CYP11A1, and HSD3B2	[140]
Cryptotanshinone (CTS)	Danshen	Roots	Suppression of cell proliferation, full-length AR transactivation, and cancer growth in xenograft PCa model	↓ AR, TMPRSS2, TMEPA1, and PSA	[141]
Epigallocatechin-3-gallate (EGCG)	Green tea	Leaves	Reduction of histone acetylation and cell proliferation in xenografts	↓ AR, nuclear translocation receptor, and NF-κB	[142]
Fisetin	Strawberries, apples, persimmons, onions, kiwi, and cucumbers	leaves, stems, and fruits	Slow down therapy resistance, invasion, and migration of cancer cells	MMP-2, MMP-9, PI3K/AKT, NF-κB pathway, AR, and PSA	[143,144]
*Garcinia mangostana/α-*Mangosteen	Purple mangosteen		Stimulation of programmed cell death, inhibition of nuclear translocation, and tumor growth in CRPC	↓ AR, AR-V7, BiP, and GRP78	[145]
Ginsenosides Rg3	Panax ginseng, Panax quinquefolius, Panax japonicus, Panax notoginseng, Panax cocos, and Pfaffia paniculata	Roots	Induction of cell cycle arrest in the G1 phase	↓ AR, AR-Vs, 5α-reductase, PSA, PCNA ↑p21, p27, caspase 3	[146]
Honokiol	Magnolia grandiflora and Magnolia dealbata.		Induction of programmed cell death, suppression of cell viability, stimulation of nuclear translocation of AR	↓ AR, AR translocation, transcriptional activity of AR	[147]
luteolin			Inhibition of cell proliferation, CRPC tumor growth, and induction of apoptosis	↓ AR-V7 via miR-8080 ↑ Sensitize enzalutamide	[148]
Quercetin			Suppression of PCa progression, increase the proportion of apoptotic cells	↓ hnRNPA1, AR-V7, AR, HSP70, PSA	[149]
Resveratrol	Grapes, Blueberries, Peanuts		Enhancement of apoptosis, slow down the growth of CRPC cells, and prevention of tumor formation in vivo	↓ PI3K/AKT, AR-V7, AR, PSA	[150]
Silibinin	Silybum marianum or milk thistle		Inhibition of cell proliferation, induction of cell cycle arrest and apoptosis in CRPC	↓ AR, PSA	[151]
Sulforaphane	Broccoli, Brussels sprouts, and cabbage	Vegetables	Suppression of cell proliferation, migration, and clonogenic potential	↓ AR-V7, HSP90 ↑ Nrf2	[152,153]
Triptolide	Tripterygium wilfordii		Induction of apoptotic cell death, inhibition of CRPC tumor growth	↓ AR, AR-V7 at Ser via XPB/CDK7, PSA/KLK3, TFIIH and RNA Pol II recruitment	[154]
Ursolic acid			Reduction of cell proliferation in vitro and xenograft tumor growth in animal models	↓ IκB kinase, IκBα, NF-κB, TNF receptor-associated factor, IkappaBalpha kinase, p65, cyclin D1, cyclooxygenase 2, and matrix metalloproteinase 9	[155]

↑, upregulated; ↓, downregulated.

## Data Availability

Not applicable.
